# Revisiting the Application of Machine Learning Approaches in Predicting Aqueous Solubility

**DOI:** 10.1021/acsomega.4c06163

**Published:** 2024-07-31

**Authors:** Tianyuan Zheng, John B. O. Mitchell, Simon Dobson

**Affiliations:** † School of Computer Science, University of St Andrews, St Andrews, Fife KY16 9SX, U.K.; ‡ EaStCHEM School of Chemistry, University of St Andrews, St Andrews, Fife KY16 9ST, U.K.

## Abstract

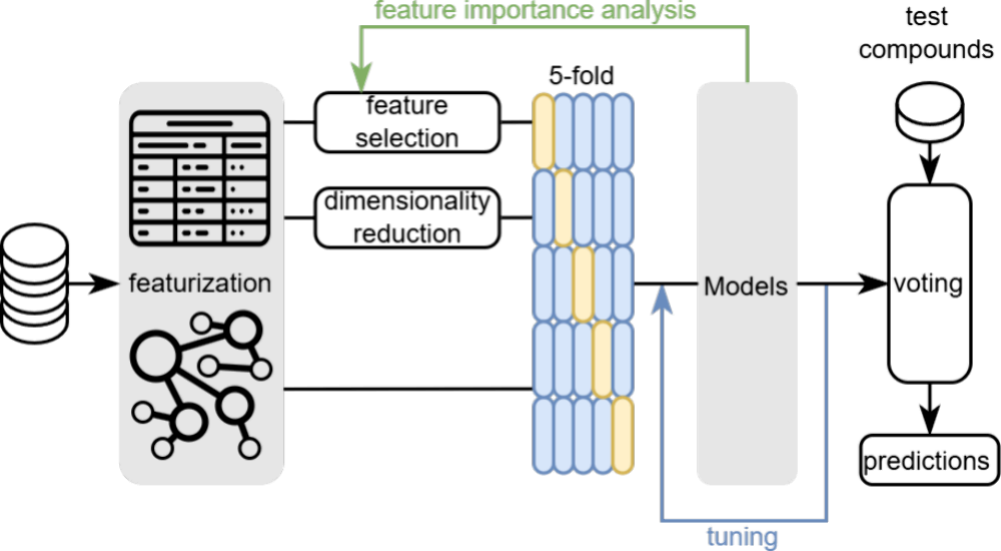

The solubility of chemical substances in water is a critical parameter in pharmaceutical development, environmental chemistry, agrochemistry, and other fields; however, accurately predicting it remains a challenge. This study aims to evaluate and compare the effectiveness of some of the most popular machine learning modeling methods and molecular featurization techniques in predicting aqueous solubility. Although these methods were not implemented in a competitive environment, some of their performance surpassed previous benchmarks, offering gradual but significant improvements. Our results show that methods based on graph convolution and graph attention mechanisms demonstrated exceptional predictive abilities with high-quality data sets, albeit with a sensitivity to data noise and errors. In contrast, models leveraging molecular descriptors not only provided better interpretability but also showed more resilience when dealing with inherent noise and errors in data. Our analysis of over 4000 molecular descriptors used in various models identified that approximately 800 of these descriptors make a significant contribution to solubility prediction. These insights offer guidance and direction for future developments in solubility prediction.

## Introduction

Solubility refers to the maximum amount of solute that can dissolve in a certain amount of solvent at specific conditions. It mainly depends on the composition of solvent and solute and is also influenced by factors such as temperature, pressure, pH levels, ionic strength, lattice energy, and many others.^1,2^ For many organic compounds such as most pharmaceutical molecules, the impacts of functional groups, molecular polarity, presence of hydrophobic groups, three-dimensional spatial structure, and conjugated systems are also important.^3−5^

The ability of a candidate drug to dissolve in water is crucial for its successful elicitation of the desired pharmacological response.^1,6−9^ Suboptimal aqueous solubility of drug molecules can lead to reduced bioavailability and therapeutic efficacy.^10−12^ According to Sharma et al.,^13^ approximately 40% of newly discovered lipophilic drug candidates do not reach the market due to their poor aqueous solubility. Such drugs with insufficient solubility may not be adequately absorbed into the bloodstream, consequently reducing their therapeutic effectiveness.^1^ Moreover, undissolved drug particles can accumulate within the body and potentially lead to blood flow obstruction, which can cause serious health issues including tissue damage and even organ failure in more severe cases.^6^

However, determining the aqueous solubility of candidate drugs is experimentally expensive. To select more promising drugs and reduce high development costs in later stages due to solubility issues, a series of predictive assessments are commonly conducted to evaluate the aqueous solubility of these candidates. These predictive techniques can be broadly grouped into two main categories: “first-principles” calculations and cheminformatics approaches. “First-principles” calculations involve computational approaches and physical models that use quantum mechanical calculations, statistical thermodynamics, or molecular simulation techniques to determine the thermodynamic properties of solute and solvent molecules, such as their energy, entropy, and solvation free energy. This approach typically does not require training data and naturally offers better interpretability. On the other hand, cheminformatics methods aim to discover the correlations between molecular properties and their solubility data, without explicitly considering the fundamental physical laws governing the dissolution process.^14^ Therefore, the accuracy and generalizability of cheminformatics models are inevitably constrained by the quality and quantity of training data, as well as the reliability of methods used to quantify and featurize molecular properties.^15^ Despite these limitations, many cheminformatics approaches offer advantages over “first-principles” methods in terms of computational cost and predictive accuracy in high-throughput solubility prediction.

Over the past two decades, classical statistical and machine learning methods have been widely used for solubility prediction, including multivariate linear regression, principal component regression, partial least-squares, *k*-nearest neighbors, support vector machines, random forest regression, and some others.^16−21^ These methods demonstrated similar levels of predictive performance and are believed to be approaching their limits in terms of accuracy and efficiency.^16,22,23^

In recent years, with the rapid development of high-performance computing hardware, deep learning (DL) methods have increasingly gained favor. Francoeur and Koes developed a solubility prediction framework called SolTranNet that is based on the molecule attention transformer.^24^ A molecular graph attention architecture named MolGAT has also been developed to predict solubility and provide insights in energy storage.^25^ Further, Cui et al. adopted a residual convolutional neural network architecture comprising approximately 20 layers, for predicting the water solubility of compounds.^26^ Panapitiya et al. evaluated various DL approaches, including SchNet based on 3D atomic coordinates, long short-term memory neural networks taking SMILES strings as inputs, graph neural networks (GNNs) with graph convolutions, and models based on molecular descriptors.^27^ A structure-aware approach has also been developed using deep network architectures and transfer learning methods to predict solubility.^28^ Apart from these, there are also a series of GNN architectures that can be applied to water solubility prediction, including directed edge graph isomorphism networks,^29^ graph-based message passing networks,^30^ and multilevel graph convolutional networks.^31^

However, the challenges in solubility prediction are far from being fully resolved.^27,32^ In this work, rather than developing new modeling architectures, we focused on evaluating and comparing the impact of some of the most popular ML methods on aqueous solubility prediction. We gathered and examined solubility data for more than 84,000 molecules, with differing levels of estimated average reproducibility across laboratories. Utilizing a range of molecular featurization techniques, we then evaluated the solubility prediction abilities of several common ML modeling methods, including graph neural network architectures, tree-boosting methods, and one-dimensional neural networks. Although not executed under competition conditions, these approaches achieve better scores in several instances than the best models available at the time, offering incremental but still significant improvements. Our results showed that while graph convolution and graph attention mechanisms display promising predictive strength with high-quality data sets, they tend to be more sensitive to noise and errors in the data. On the other hand, models that leverage molecular descriptors offer better interpretability and show better resilience to noise and inaccuracies in the data set. We carried out an analysis of over 4000 molecular descriptors used in different models, finding that about 2000 were effective, with roughly 800 making significant contributions to aqueous solubility prediction.

### Data

Our prediction of aqueous solubility is divided into regression and classification problems. Compounds in these problems are provided in the SMILES (Simplified Molecular Input Line Entry System)^33^ format, a standardized method of representing the chemical structures of molecules using ASCII strings.

In regression problems, aqueous solubility data of compounds is represented by log *S*, the base-10 logarithm of a compound’s solubility in water, where *S* is measured in moles per liter. The training data for the regression problems is prepared from the following sources. (i) 2008 Solubility Challenge training set,^34,35^ 2019 Solubility Challenge training set,^16,32^ and the DLS-100 data set,^36−38^ with data assessed using the “Chasing Equilibrium” technique (CheqSol).^39^ (ii) AqSol data set^40^ which combines nine data sets from various source. (iii) AQUA data set obtained from Meng et al.,^41^ containing research data by Huuskonen^42^ and Tetko et al.,^43^ with experimental aqueous solubility values measured between 20 to 25 °C, sourced partly from the AQUASOL database of the University of Arizona and SCR’s PHYSPROP database. (iv) PHYS data set^41^ obtained from Meng et al.,^41^ containing molecules with water solubility end points extracted from the PHYSPROP database. (v) Some other curated solubility data.^44−51^

These data sets use different assays with varying precision and sensitivity, inevitably leading to systematic biases in solubility values and thus introducing noise. Correspondingly, one of the objectives of this study is to investigate the tolerance of different ML algorithms to this noise, comparing the performance of models when handling data of varying sources and quality.

**Figure 1:**
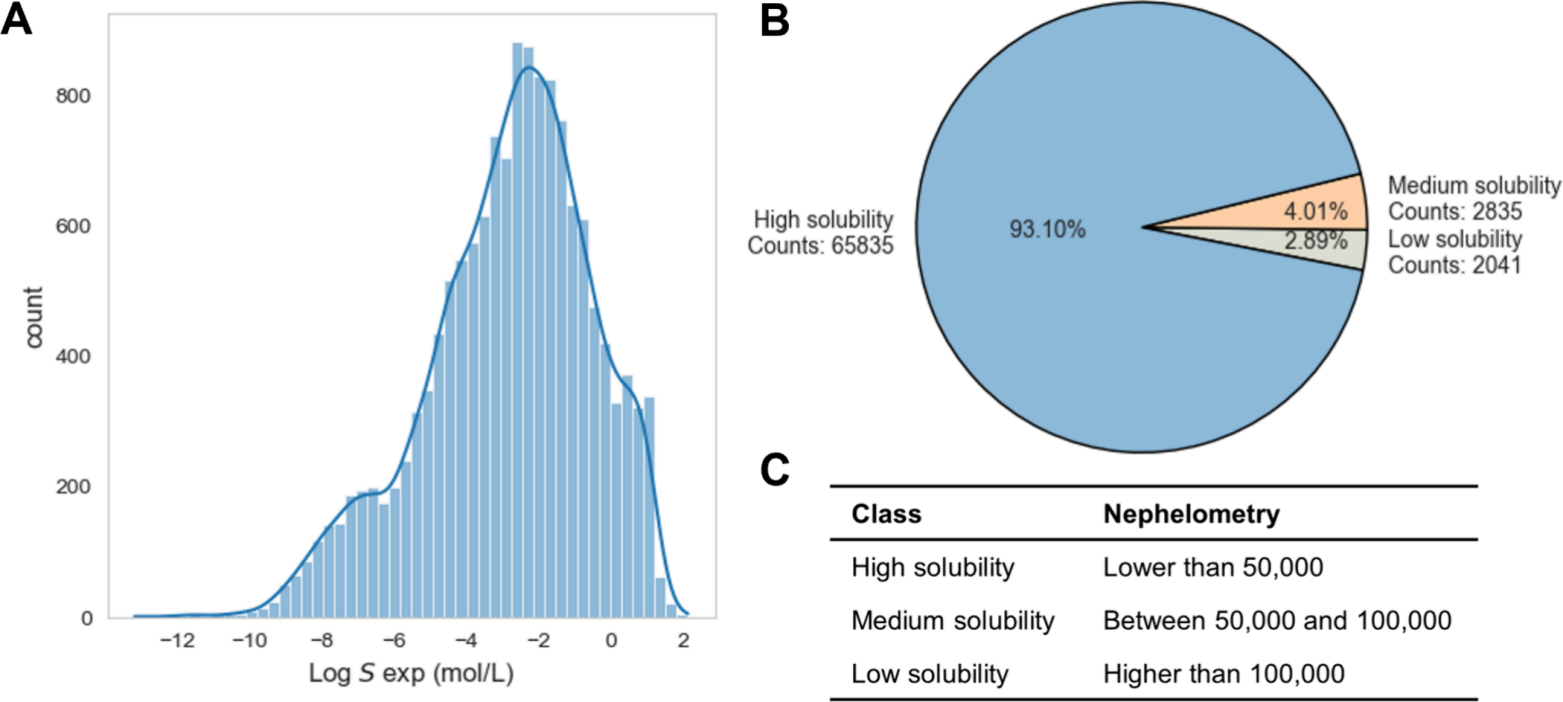
(A) Distribution of log *S* values in the training data prepared for regression problems. (B) Distribution of classes and (C) classification criteria in the imbalanced training data from the EUOS/SLAS Solubility Prediction Challenge.

To evaluate the performance of models trained on this data set, the test data comprises druglike compounds from the 2008 Solubility Challenge test set [08SC; 28 compounds; the estimated average interlaboratory reproducibility for the molecules (AIR) ≈ 0.05]^34,35^ and the 2019 Solubility Challenge test sets 1 (19SC1; 89 compounds; AIR ≈ 0.17) and 2 (19SC2; 26 compounds; AIR ≈ 0.62).^16,32^ According to Llinas et al.,^32^ 19SC2 includes a selection of “contentious” molecules characterized by having a higher average uncertainty in their solubility measurements compared to 19SC1. The training and test sets were carefully checked for duplicates, and all such instances were removed, resulting in 14,432 compounds, with their solubility distribution shown in Figure 1A.

The data set for classification tasks is collected from the first EUOS/SLAS Joint Solubility Prediction Challenge,^52^ consisting of 70,710 training and 30,307 testing samples. In this data set, aqueous solubility is determined using nephelometry, a technique that detects the scattering of light as a laser beam passes through a suspension.^53−55^ It is not designed to provide precise quantitative solubility measurements but serves as a protocol for “quick-and-dirty” assessing of the kinetic solubility of a large compound data set.^53^ Compounds are then categorized into three groups based on their nephelometry results (Figure 1C). Additionally, imbalance is a key attribute of this training data set (Figure 1B), with 93.1% of compounds being classified under the “high solubility” category.

### Featurization

Most ML algorithms are not designed to directly interpret the textual representations of SMILES and their structural information about molecules. On the basis of the specific demands of the chosen model, we transformed SMILES into a representation that is compatible with the model’s specific characteristics.

Molecular descriptors are numerical representations that quantify the structure and properties of molecules. Thousands of descriptors can be used to encode molecules, ranging from ones derived solely from the chemical structure to experimentally derived quantities.^37^ Specifically, we used the following tools to featurize SMILES into molecular descriptors: Mordred,^56^ RDKit,^57^ Extended-Connectivity Fingerprints (ECFP),^58^ PubChem,^59^ Mol2Vec,^60^ Optimized MDL,^61^ and CDK.^62^ In total, 4480 descriptors were considered in our study.

Molecular structures can also be naturally represented in graphical form. A graph typically consists of nodes interconnected by edges. Nodes can encapsulate a range of attributes, including but not limited to atom type, degree, number of implicit hydrogen atoms, and formal charge. Edges, on the other hand, may carry features such as bond types, conjugation status, ring involvement, and directional attributes of the bond. On the basis of the form or characteristics of the edges, such graphs can be categorized into various types, including cyclic graphs, directed graphs, and undirected graphs. Such graph representations are usually described using mathematical structures such as adjacency matrices or edge lists, making them well-suited as inputs for GNNs. In our study, we used DGL,^63^ DGL-LifeSci,^64^ and PyTorch Geometric^65^ for featurizing molecular structures into their corresponding graph representations and utilized these representations for constructing various GNNs to extract features and identify underlying patterns.

### Dimensionality Reduction for Data Insights

Different features in a data set often come with varying scales and units. If some features in the data have larger numerical ranges, they can disproportionately dominate a model’s outcome because features having smaller scales might be equally or more relevant to the underlying patterns in the data.^66−68^ We normalized feature values to ensure they are on a comparable scale and carry equal weight during model training.

Inputting all features directly into the ML model is generally not a good idea. In situations where the number of training samples is constant, increasing the number of features initially improves the performance of models, but after reaching a certain point, further increases in dimensions lead to a decrease in performance.^69^ As dimensions increase, the data becomes sparser in the multidimensional space, inevitably requiring more data to achieve meaningful statistical insights, resulting in higher computational costs.^70^

**Figure 2:**
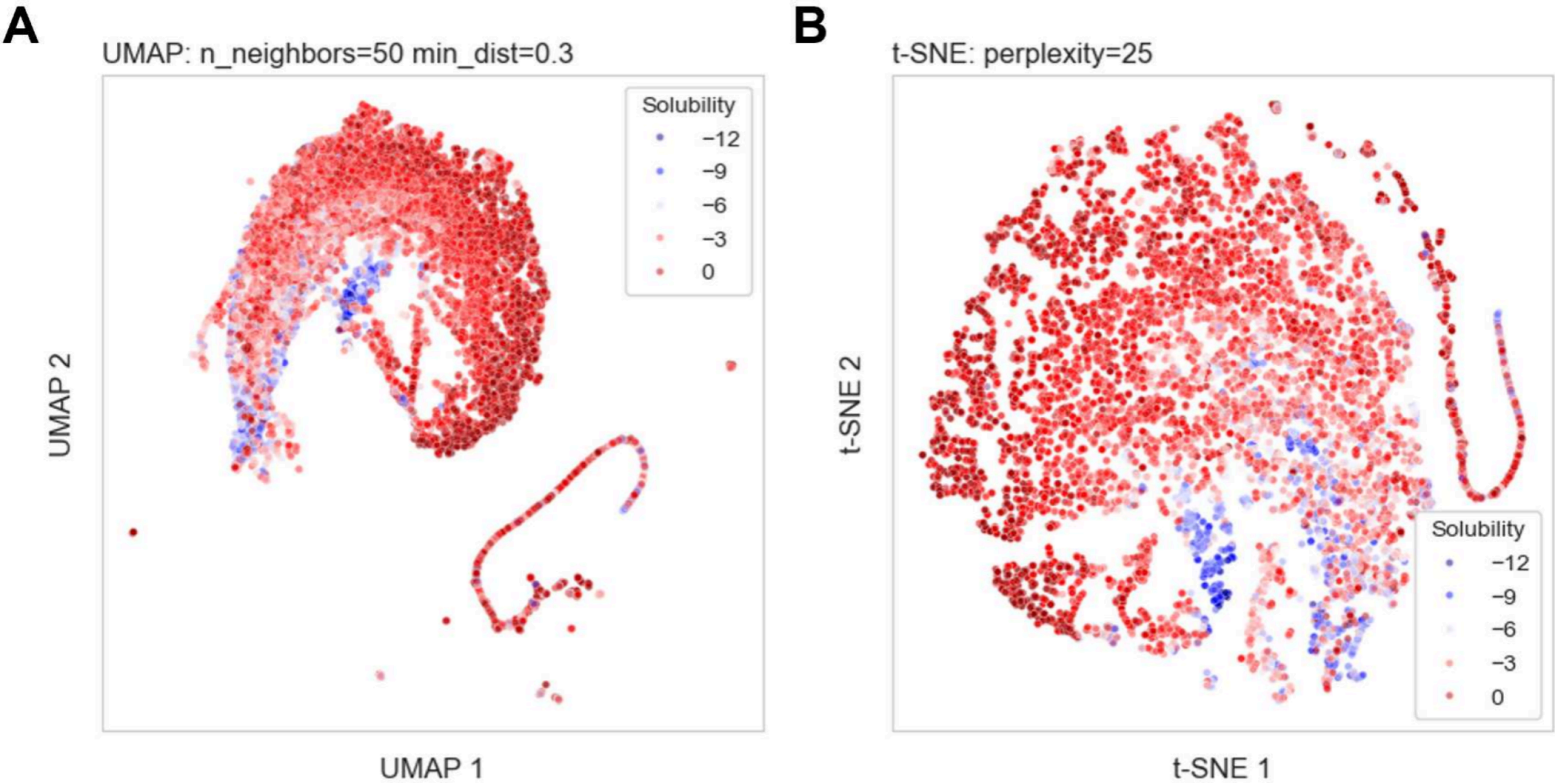
Projection of high-dimensional training data prepared for regression problems into a two-dimensional space using (A) UMAP and (B) t-SNE.

To preliminarily explore the structures and patterns within high-dimensional data, we used two nonlinear dimensionality reduction techniques, t-SNE (t-Distributed Stochastic Neighbor Embedding)^71^ and UMAP (Uniform Manifold Approximation and Projection)^72^ to embed high-dimensional data into two or three-dimensional spaces for a intuitive data visualization, as shown in Figure 2A,B. We observed that t-SNE preserves the local similarity between data points, but it lacks the ability to infer global structures as effectively as UMAP. Additionally, t-SNE’s *O*(*n*^2^) time and space complexity^71^ make it both time-consuming and memory-intensive when dealing with large data sets. In contrast, UMAP, a much more efficient option, captures both the local variations and the overall layout and relationships within the data. In Figure 2A, the low-dimensional projections of compounds are arranged in a bandlike formation, with a decrease of solubility gradient from the head to the tail of the band.

### Evaluation Criteria

Root mean squared error (RMSE) and coefficient of determination (*R*^2^) are used as performance evaluation metrics for models’ ability to predict the continuous variable log *S*, with their definitions given as follows:
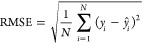
1
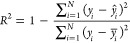
2where ŷ is the predicted log *S* value, *y* is the literature log *S* value, y̅ is the mean of literature log *S* values, and *n* is the number of samples.

For the imbalanced classification problems, relying solely on metrics such as accuracy and even the confusion matrix can prove inadequate. Quadratic Weighted Cohen’s Kappa (QWK) is a more appropriate metric for imbalanced classification problems, where misclassifying certain classes may have more significant implications than others. This was the metric specified for the EUOS/SLAS competition.^52^ The mathematical expression for QWK is as follows:
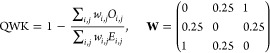
3where *O* is the histogram matrix and *E* is the expected matrix.

### Aqueous Solubility Prediction Pipeline

**Figure 3:**
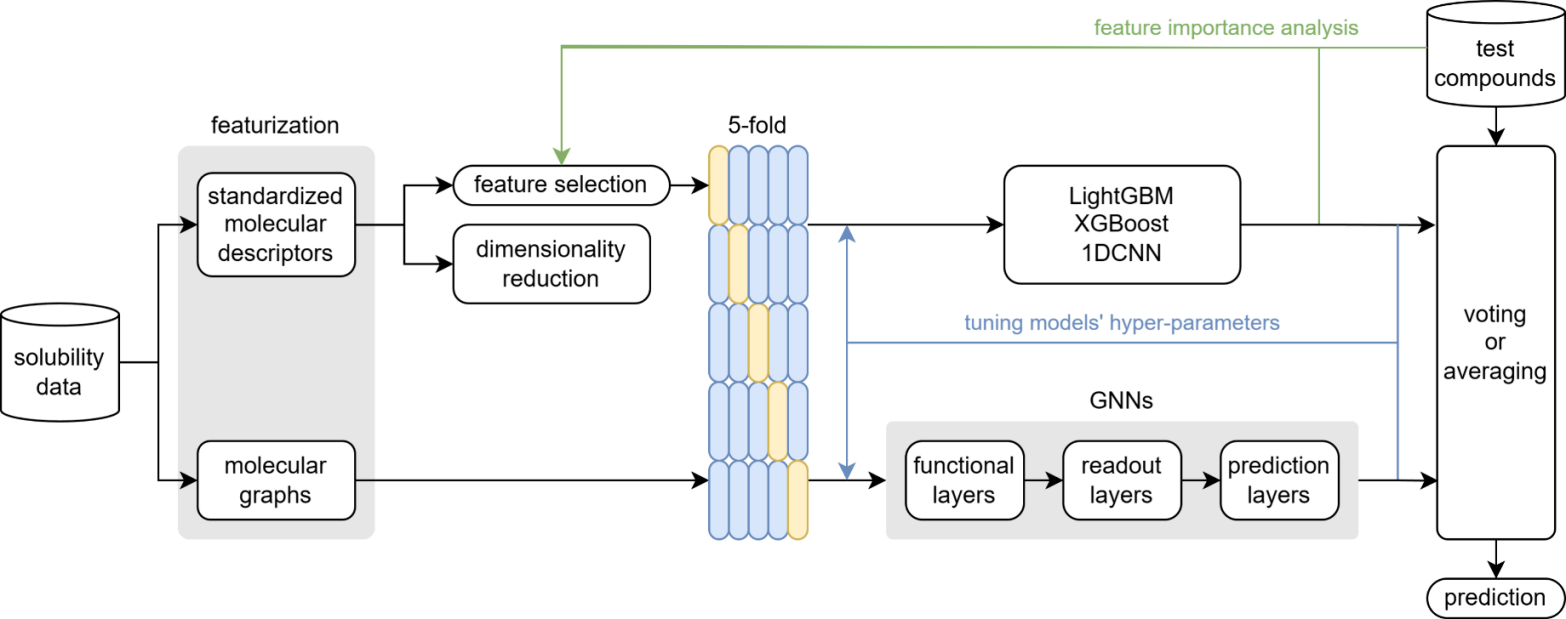
A schematic representation of the training, prediction, and iterative processes in the solubility prediction pipeline.

Figure 3 gives an overview of the training, prediction, and iterative processes involved in the solubility prediction. After removing duplicate compounds and compounds with missing values, the solubility data were featurized into representations that could be processed by ML models; then dimensionality reduction was conducted on these standardized molecular descriptors before training to gain more insights, as described above. Considering that suboptimal performance of ML models does not always indicate a flaw in this model, we used 5-fold cross-validation (CV) to assess the current configured model’s performance.

Independently from this blind CV performed, in the actual prediction stage, we conducted regular performance evaluations to monitor the model’s progress in real-time and guide the selection of retrospective model checkpoints. These evaluations were typically based on training steps rather than time intervals. Each training epoch implemented an early stopping mechanism, whereby training would be terminated if there was no improvement in validation performance over 20 epochs.

For classification tasks, graph-based models and 1DCNN generate probabilities for the three classes, and the class with the highest probability is selected as their prediction; XGBoost and LightGBM use their built-in multiclass methods to directly output the predicted class. For regression tasks, all models output a continuous value. In the final prediction stage, regardless of whether the task is classification or regression, five models are trained on newly sampled 5-fold data that is independent of the previous blind CV. The final prediction is then obtained by averaging the predictions of these *k* models for regression tasks or by majority voting for classification tasks.

The high-level structure of graph-based models consists of a sequence of components: functional layers that extract initial node features, readout layers that aggregate these features into a fixed-size graph representation, and prediction layers that make the final predictions. More details will be described later.

Even the comparison of optimizer performance presents a challenging task.^73^ We opted for an established and popular optimizer, Adam,^74^ which is more versatile than stochastic gradient descent with momentum.

For descriptor-based models, considering that not all generated descriptors equally benefit the final solubility prediction, and to further mitigate the curse of dimensionality as well as reducing noise and redundancy, we employed Permutation Variable Importance^75^ and Shapley Additive Explanations (SHAP) feature importance^76,77^ to evaluate the contribution of each descriptor to the final prediction after the training session. We then selected some subsets of the most significant descriptors for predicting solubility and compared the impact of these subsets of varying sizes on the overall model performance.

We also employed quasi-random search algorithms to tune the hyper-parameters of our models, which allows us to progressively narrow the search space while also ensuring uniform sampling of hyper-parameter values. Once we have finished exploring the approximate search space and identifying the hyper-parameters that required finer tuning, our focus shifted from an in-depth understanding of the tuning issue to an optimal configuration for deployment. As our goal was no longer centered on maximizing our knowledge of the tuning problem, the numerous advantages of quasi-random search became less pertinent at this stage.^78^ In the subsequent training and validation phases, we transitioned to using Bayesian optimization tools, which are adept at automatically discovering the best hyper-parameter configurations.

### Computational Methods

#### Extreme Gradient Boosting

Extreme Gradient Boosting (XGBoost) is a scalable end-to-end tree boosting system^79^ that uses weighted quantile sketch for fast approximate tree splitting and a sparsity-aware algorithm for parallel tree learning. It adopts the Newton–Raphson method in function space, using second-order derivatives for more precise parameter updates, rather than the traditional gradient descent. XGBoost also handles sparse input features and missing values by learning the optimal strategy based on the training loss, leveraging sparsity to achieve a linear computational complexity with respect to the number of nonmissing entries in the input.

#### Light Gradient-Boosting Machine

Similar to XGBoost, Light Gradient Boosting Machine (LightGBM),^80^ a gradient boosting framework originally developed by Microsoft, is based on gradient-boosted decision tree algorithms. They share many advantages and features, such as optimizations for sparse data. One of the main difference between LightGBM and XGBoost is their tree-growing strategies. Unlike XGBoost, which uses a level-wise growth strategy, LightGBM expands each leaf yielding the maximum reduction in loss. Moreover, LightGBM handles data with a histogram-based algorithm which stands in contrast to the presorted and approximate algorithms used by XGBoost.

#### 1D Convolutional Neural Network

In deep two-dimensional convolutional neural networks, convolutional kernels move across two-dimensional spaces to capture the spatial features of input data. However, 2DCNNs may not be applicable with one-dimensional input signals. The one-dimensional convolutional neural network (1DCNN) has been proposed, in which the convolutional kernels slide along the sequence dimension of the data to capture local features in one-dimensional data. It has also demonstrated some exceptional performance across various domains.^81−84^

**Figure 4:**
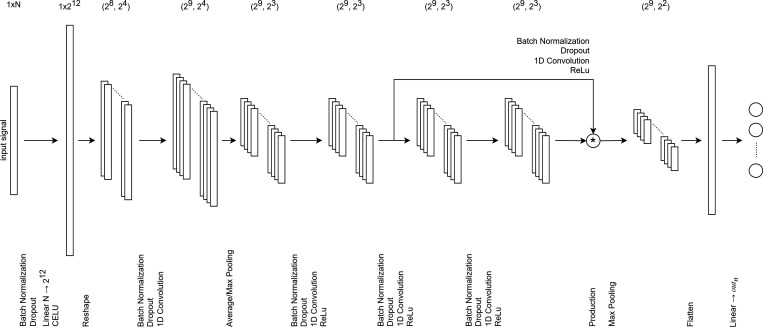
A fully connected neural network with 1D convolutions and shortcuts.

Figure 4 illustrates the fully connected neural network with 1D convolutions that we used for solubility prediction when the number of input features exceeded 3000. The network begins with a dense layer that normalizes, drops, and linearly transforms the input features, setting up a foundational feature space. It then progresses through a series of convolutional layers, with each of these layers incorporating batch normalization, dropout, and ReLU activation to further refine the features. A key aspect of this architecture is the shortcut connections, where outputs from certain layers are stored and later combined with outputs from subsequent layers. This blending of features helps the network in preserving and emphasizing important characteristics of the data. The decoder, which includes a max pooling step that reduces spatial dimensions by selecting the maximum value within nonoverlapping subregions of its input, is followed by flattening, batch normalization, dropout, and a final linear layer, compresses and maps features to the desired output space.

#### Graph Neural Networks

GNNs, which can be considered as extensions of recurrent neural networks and random walk models, are designed to process structured data represented in the form of graphs. The core of GNNs in processing graph data is message passing. Each node uses its own feature information and aggregates information from its neighboring nodes to update its representation in each iteration, such that the network itself can learn complex patterns and relationships within the graph.

In this study, we abstract the architectures of various GNNs we explore into a combination of three types of layers: functional layers, readout layers, and prediction layers, as shown in Figure 3. The functional layers, potentially comprising one or multiple concrete layers, process the information derived from the nodes and edges within the graph by aggregation or message passing. The specific implementations of these operations vary depending on the particular GNN algorithm as detailed below, but their shared objective is to capture relationships between nodes while preserving individual node characteristics. Then, the readout layer generates a global representation of the entire graph based on the node features learned by the functional layers. Finally, the prediction layers are a series of fully connected layers that further process and transform the outputs obtained from the functional and readout layers. These layers are where the final decision-making or forecasting takes place.

##### Graph Convolutional Network

The Graph Convolutional Network (GCN) is a variant of convolutional neural networks (CNNs) that operates directly on graph-structured data.^85^ This form of GNN extends the concept of convolution from the Euclidean spaces typical in traditional CNNs to non-Euclidean spaces. It is capable of handling the varied and unordered connection patterns found among nodes in graphs.

In the graph convolutions within a GCN, the new feature representation of a node is updated by applying a nonlinear transformation to the weighted combination of its own features and those of its neighboring nodes. As discussed by Kipf and Welling,^85^ the layer-wise propagation rule of GCN is defined as
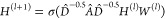
4where *H*^(*l*)^ is the feature matrix of nodes at the *l*th layer,  is the adjacency matrix *A* of the graph with added self-loops, denoted by the identity matrix *I*_*N*_,  is the degree of node *i* including self-loops, *W*^(*l*)^ is the weight matrix for the *l*th layer, and σ is the activation function.

In our GCN model, the functional layer comprises three graph convolutional layers with dimensions 256, 128, and 64, respectively. Following each convolutional layer, a dropout layer is applied to prevent overfitting and to enhance the model’s generalization ability. Due to the inherent design constraints of GCNs, the model’s inputs are restricted to undirected graphs and users are unable to use edge featurization techniques as part of the molecular graph representation. Furthermore, it implicitly assumes locality (dependence on the *K*th-order neighborhood for a GCN with *K* layers) and equal significance of self-connections and edges to neighboring nodes.

##### Graph Attention Network

The Graph Attention Network (GAT)^86^ is a GNN architecture which integrates attention mechanisms to dynamically weight connections between nodes. In GAT, attention scores determine the extent to which neighboring nodes influence the feature update of a given node, allowing the model to focus more on the most important neighbors. Unlike GCN, GAT does not rely on a fixed graph structure. Instead, it dynamically learns the strength of relationships between nodes to adapt to irregular graph structures.

Each attention layer in a GAT consists of multiple attention heads, with each head performing independent graph convolution operations to capture the relationships between nodes from various perspectives. The diverse feature representations obtained from these heads are then combined, either by concatenation or averaging, to form the final feature representation of each node.

As per Veličković et al.,^86^ the propagation rule for a single layer in a GAT is defined as
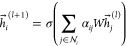
5where  is the feature vector of node *i* at the *l*th layer,  is the set of neighboring nodes of *i*, and α_*ij*_ is the attention coefficient that measures the importance of node *j* to node *i*.

In our implementation, we stacked two attention layers with sizes of 128 and 64 as the functional layers, each equipped with five attention heads.

##### Graph Attention Network Version 2

A key limitation of GAT is the static nature of its attention mechanism: attention scores remain fixed and independent of the querying node, which curtailed the model’s ability to accurately represent and learn from training data.^87^

Graph Attention Network version 2 (GATv2)^87^ proposed by Brody et al. is an enhanced version of GAT. GATv2’s attention heads dynamically adjust the attention scores to mitigate the effect of GAT’s static nature: the impact of neighboring nodes on a target node can dynamically change in response to variations in node features. In our implementation, we stacked two attention layers with sizes of 128 and 64, respectively, each with five of these dynamic attention heads.

##### Message Passing Neural Network

The Message Passing Neural Network (MPNN)^88^ introduced by Gilmer et al. is a GNN architecture that was initially designed for addressing quantum chemistry problems. Operating on undirected graphs with both node and edge features, MPNN abstracts the spatial convolutions and can serve as a universal framework for spatial-based GCN.^89^ Unlike simple neighborhood aggregation strategies, the node updating rules in MPNN operate through the receipt and processing of messages from neighboring nodes. Specifically, in the message passing phase executing over *t* = 1, ..., *T* time steps, hidden states *h*_*v*_^*t*^ at each node in the graph are updated according to
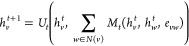
6where *M*_*t*_ is the message function that computes the messages exchanged between nodes at *t*, *U*_*t*_ is the vertex update function that updates the state of each node based on the aggregated messages received from its neighbors, and *e*_*vw*_ represents the edge features between nodes *v* and *w*.

##### AttentiveFP

In the propagation process of networks such as GCN, the influence of neighboring nodes on a target node decreases with their increasing topological distance. However, in molecular graphs, topologically distant atom pairs can have significant interactions, since the corresponding atoms could be spatially close or might be part of the same delocalized system. In contrast, networks such as MPNN construct virtual edges between every pair of nodes in molecular graphs, which makes sure that any node, regardless of its distance from the target node, has an equal opportunity to influence any other. But in molecular structures, adjacent nodes often have stronger interactions, especially when forming functional groups.^90^

AttentiveFP^90^ developed by Xiong et al. is a GNN architecture designed for drug discovery and molecular property prediction. Unlike general GNN architectures such as GCN and MPNN, AttentiveFP incorporates molecular fingerprinting techniques and focuses on modeling molecular data and chemical characteristics. Furthermore, it captures local atomic environments through information propagation from adjacent to distant nodes and uses graph attention mechanisms to account for nonlocal interactions within molecules.

## Results and Discussion

### Descriptor Importance

For each ML method that takes molecular descriptors as inputs, we employed the 5-fold CV approach as previously described, wherein five separate instances of each method were trained across five unique combinations of training and validation data sets. Subsequently, the contribution of each molecular descriptor to the predictive capability across the models was evaluated using the union of three independent regression test sets (see Supporting Information 1).

**Figure 5:**
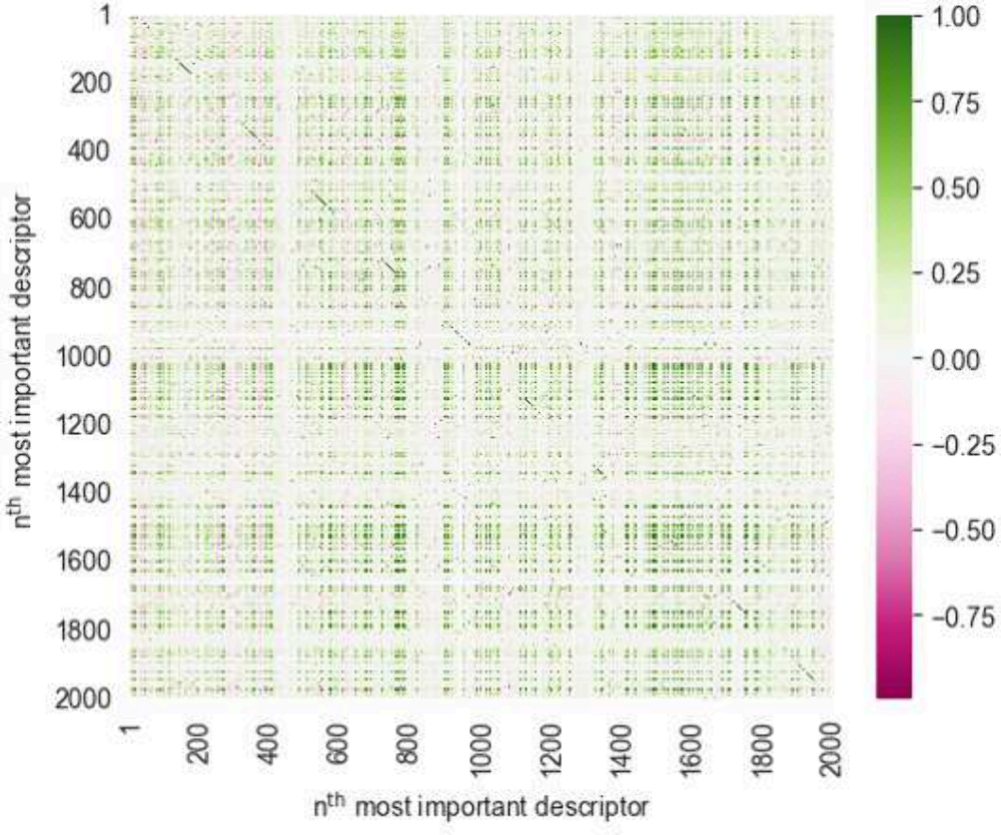
Linear correlation among the top 2000 most important features.

Different models learn and process features in different ways, thus the importance of features varies depending on the choice of the model. Despite this variability, we have still observed a similar trend in the ranking of certain features’ importance across these models. Well-trained boosting tree methods, XGBoost and LightGBM, only used about 2000 features for splitting decision trees, with the linear correlations among these features being shown in Figure 5. These two methods both show robustness against feature redundancy, as a subset of feature pairs exhibit strong positive or negative correlations. These correlated features may carry similar information, contributing overlapping or analogous influences on model predictions. Upon further analysis of these 2000 features’ contributions to the predictions, it is found that approximately 800 features have a more conspicuous impact on the model’s decision-making process, including the following. (i) Thirty five descriptors linked to log *P*. The log *P* value indicates a compound’s distribution coefficient between two distinct solvents, octanol and water, so these log *P*-related descriptors are useful for assessing the hydrophobic and hydrophilic characteristics of compounds. This category includes descriptors such as JPLogP, MolLogP, XLogP, FilterItLogS, ALogP, PLogP, SLogP, and MLogP. (ii) Approximately 170 descriptors related to the electronic distribution and charge states of molecules, for example: the relative negative charge descriptor (RNCG) and relative positive charge descriptor (RPCG) quantify the relative size and significance of negative or positive charge regions within the molecule; E-State VSA descriptors combine electrotopological indices and molecular surface area contributions; MaxAbsPartialCharge describes the maximum absolute partial charge within the molecule. (iii) Approximately 500 descriptors calculated based on the topological characteristics of molecules, involving the relative positions and connectivity of atoms within the molecule. A prime example is the Autocorrelation of Topological Structure (ATS) descriptor, also known as the Moreau-Broto autocorrelation descriptor. This descriptor evaluates the correlation between specific properties of atom pairs (such as charge or mass) and their topological distance (the number of bonds separating them), to characterize the molecular structure. Related descriptors include AATS (Averaged ATS), ATSC (Centered ATS), and AATSC (Averaged ATSC). Among these, descriptors such as AATSC 1s, ATSC3i, and ATSC6pe stand out for their high importance scores. (iv) The Quantitative Estimate of Drug-likeness (QED), a metric which quantifies the drug-likeness of compounds based on a variety of their physicochemical properties, including molecular weight, log *P*, the number of hydrogen bond donors and acceptors, the polar surface area, and many others. (v) Other topological descriptors, such as the number of bridgehead atoms, the count of basic nitrogen atoms in a molecule and circular fingerprints, such as ECFPs, that are designed to capture molecular features relevant to molecular activity.

**Figure 6:**
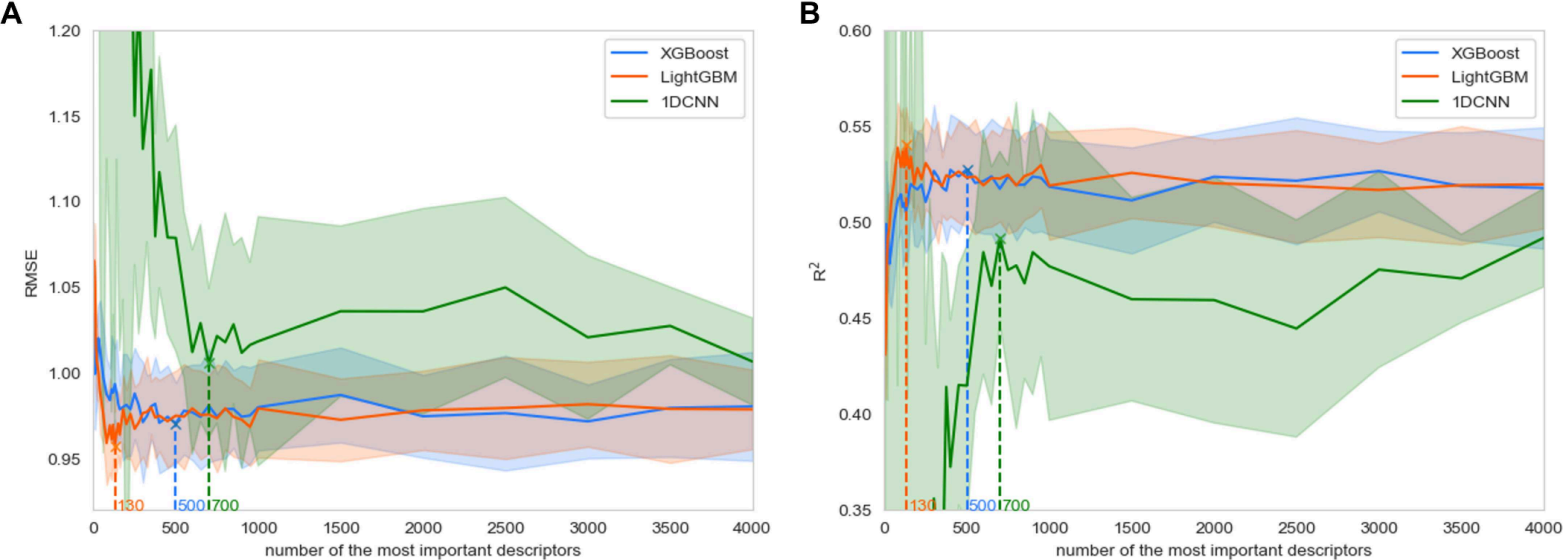
Evaluating the impact of feature selection on the predictivity of three models in line charts with error bars representing standard deviation, measured in terms of (A) RMSE and (B) *R*^2^ across various sizes of optimized feature subsets, tested on the union of three independent test sets (08SC ∪ 19SC1 ∪ 19SC2). Cross marks and dashed lines indicate the number of most important features in the feature sets when each model achieves its optimal predictive capability.

To reduce the sparsity of the training data and identify the minimal set of features necessary for sufficiently satisfactory predictive performance of the model, we conducted additional 5-fold CV. This process was aimed at assessing the model’s performance with varying numbers of important features. Specifically, we first normalized the feature importance scores calculated by different algorithms on the previous feature set. On the basis of the average importance of these features, we then ranked and selected the top-ranking features. This procedure was performed iteratively. Finally, we trained models using these subsets of varying sizes and recorded their predictive performance, as illustrated in Figure 6.

Before reaching the threshold of 700 selected features, an increase in the number of chosen features was observed to gradually reduce the RMSE scores while correspondingly increasing the *R*^2^ scores. For tree-based boosting methods, selecting more than 500 of the most important features did not significantly improve the models’ predictive performance. Theoretically, when the training data contain less noise, or when the training and testing data exhibit similar patterns, or when the data set size is limited, the number of required features can be further reduced because some important features are still highly correlated. However, on the other hand, using fewer descriptors may cause information loss or expose the biases inherent in these descriptors. For the 1DCNN model, although adding more features did not significantly improve its predictive performance, using the entire set of features enhanced the stability. If reducing the number of features were one’s goal, a significant reduction could probably be made with only a modest cost in terms of model performance or stability. The extent to which this is possible will vary between the model architectures, and our focus on comparing these architectures is another reason why we chose not to push feature reduction any further.

### Comparison of ML Methods

Table 1 summarizes and compares the results from ten times 5-fold CV and an extra 50 individual predictions on various test sets for different ML methods, with the detailed outcomes of each run documented in Supporting Information 2. The predictions from single instance runs of these ML methods, plotted against the solubility values sourced from literature, are depicted Figures S3.1–S3.4.

**Table 1:** Summary of Results, Represented As Mean ± SD of 50 Runs (Ten Runs for CV), in Terms of RMSE, *R*^2^, and QCK

	CV^a^		19SC1^b^		19SC2^c^		08SC^d^		EUOS/SLAS^e^	
method	RMSE ± SD	R^2^ ± SD	RMSE ± SD	R^2^ ± SD	RMSE ± SD	R^2^ ± SD	RMSE ± SD	R^2^ ± SD	QCK^f^	QCK^g^
XGBoost	0.69 ± 0.05	0.88 ± 0.02	0.80 ± 0.01	0.47 ± 0.01	1.40 ± 0.02	0.51 ± 0.01	0.89 ± 0.02	0.57 ± 0.02	0.135	0.111
1DCNN	0.71 ± 0.06	0.87 ± 0.02	0.71 ± 0.01	0.58 ± 0.01	1.54 ± 0.03	0.41 ± 0.02	0.92 ± 0.03	0.54 ± 0.03	0.111	0.121^I^
LightGBM	0.57^I^ ± 0.06	0.92^I^ ± 0.02	0.83 ± 0.01	0.43 ± 0.01	1.39^I^ ± 0.02	0.52^I^ ± 0.01	0.79^I^ ± 0.01	0.66^I^ ± 0.01		
GCN	0.59 ± 0.08	0.91 ± 0.03	0.70^I^ ± 0.02	0.59^I^ ± 0.02	1.62 ± 0.04	0.35 ± 0.03	0.89 ± 0.04	0.57 ± 0.03	0.141^I^	0.090
GAT	0.70 ± 0.10	0.88 ± 0.03	0.72 ± 0.05	0.57 ± 0.07	1.59 ± 0.06	0.37 ± 0.06	0.79^I^ ± 0.10	0.66^I^ ± 0.09	0.085	0.081
GATv2	0.67 ± 0.11	0.89 ± 0.04	0.81 ± 0.07	0.47 ± 0.11	1.43 ± 0.03	0.51 ± 0.13	0.80 ± 0.06	0.60 ± 0.05		
MPNN	0.93 ± 0.09	0.78 ± 0.04	0.96 ± 0.06	0.25 ± 0.10	1.76 ± 0.09	0.23 ± 0.08	1.08 ± 0.10	0.36 ± 0.12		
AttentiveFP	0.68 ± 0.07	0.88 ± 0.02	0.87 ± 0.09	0.37 ± 0.13	1.59 ± 0.09	0.37 ± 0.08	0.85 ± 0.10	0.60 ± 0.09	0.074	0.061
min	0.57^I^	0.78	0.70^I^	0.25	1.39^I^	0.23	0.79^I^	0.36	0.074	0.061
max	0.93	0.92^I^	0.96	0.59^I^	1.76	0.52^I^	1.08	0.66^I^	0.141^I^	0.121^I^
*ref*^h^			0.76	0.64	1.08	0.75		0.650	0.147	0.116

a Results of the model’s 5-fold blind CV on the training data set.

b 2019 Solubility Challenge test set 1.^16,32^

c 2019 Solubility Challenge test set 2.^16,32^

d 2008 Solubility Challenge test set.^34,35^

e First EUOS/SLAS Joint Challenge test set.^52^

f The public scores that are computed with approximately 50% of the test data, but which are visible throughout the competition and hence prone to overfitting.

g The private scores reflecting the final standings are computed with the remaining solubilities, again comprising approximately 50% of the test data.

h The score of the winning solution announced by the competition organizers.^32,35,52,91,92^

I The best results.

Among the descriptor-based approaches, 1DCNN excelled over tree-boosting methods XGBoost and LightGBM for 19SC1, achieving performance metrics that exceeded the highest reported in the 2019 Solubility Challenge. On the other hand, LightGBM demonstrated the most robust performance in CV and emerged as the top performer in both 08SC and 19SC2, surpassing the benchmarks in the Solubility Challenges of both 2008 and 2019.

Turning to neural network methods based on molecular graphs, MPNN failed to demonstrate a significant advantage. In contrast, methods involving graph convolution (GCN) and graph attention mechanisms (GAT) showed promising results across the 19SC1, 19SC2, and 08SC test sets, particularly in 19SC1 and 08SC where both RMSE and *R*^2^ values of predictions exceeded the benchmarks set in these years’ challenges. Interestingly, GAT, GATv2, and GCN, which do not directly consider edge features, outperformed AttentiveFP that does. There are two possible explanations for this counterintuitive observation: (1) existing algorithms for computing edge features might not effectively improve solubility prediction. The representations of chemical bonds between atoms in a graph could possibly lead to incomplete or incorrect molecular structures. (2) Node features alone might already be sufficient to capture and integrate key topological information, potentially outweighing the advantages brought by incorporating edge features.

More complex modeling methods such as MPNN, AttentiveFP, and GATv2 tend to show bigger variability in their performance across different runs even on the same test set. These models generally have more parameters and are highly nonlinear, which means they can have more flexibility but also tend to be more sensitive to how they are trained and set up. They also incorporate more random steps which can lead to varying results in each run. Taking GATv2 as an example, its dynamic attention mechanism causes its results to vary significantly between runs.

**Figure 7:**
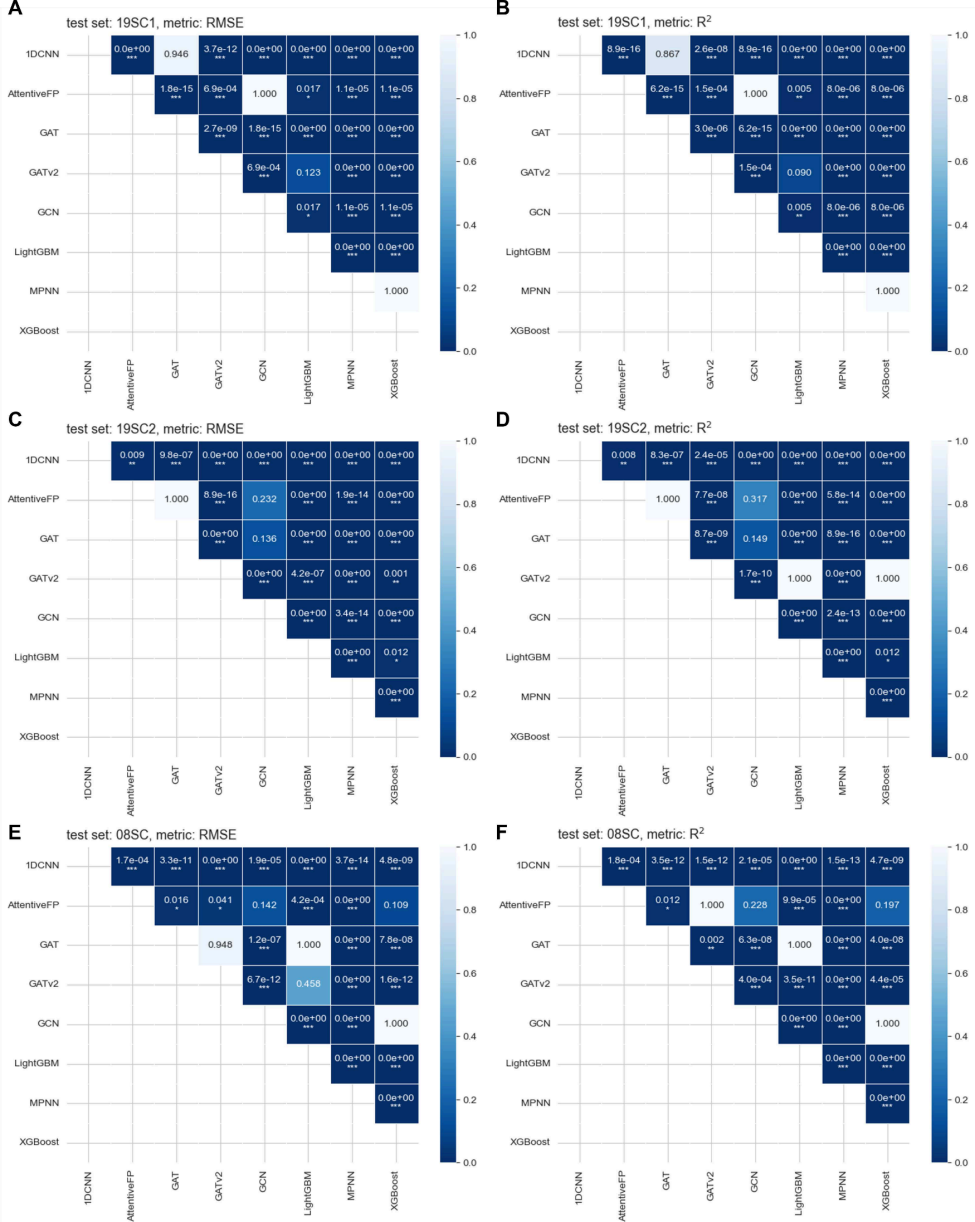
Significance of differences in (A, C, and E) RMSE and (B, D, and F) *R*^2^ values for the predictions of various ML methods on the (A, B) 19SC1, (C, D) 19SC2, and (E, F) 08SC test sets. Note that *, **, and *** indicate increasing levels of statistical significance, with * being *p* < 0.05, ** being *p* < 0.01, and *** being *p* < 0.001. When the *p*-value for a pair of methods exceeds 0.05, the null hypothesis cannot be rejected, indicating no significant difference in predictive performance between the two methods.

We then conducted a statistical analysis on the 50 prediction results from various ML methods across multiple test sets, as recorded in the Supporting Information 2, to evaluate the significant differences in these methods’ performance. The predictions’ RMSE and *R*^2^ values tended to follow a skewed normal distribution (Figure S3.5–S3.7). Upon applying a homogeneity of variance test, we observed inconsistencies in variances among different methods (Table S3.1). Therefore, we performed the Brown-Forsythe ANOVA test, which is apt for data with skewed distributions and unequal variances, showing significant statistical differences (*p* < 0.001) in the average RMSE or *R*^2^ values of at least one group of ML methods’ predictions (see also Table S3.2). To further probe the differences in predictive performance between these methods, we subsequently conducted Tamhane’s T2 posthoc test, the results of which are illustrated in Figure 7.

The differences in predictive performance between AttentiveFP, which incorporates graph attention and convolution mechanisms, and other graph-based methods like GAT, GATv2, and GCN were not particularly significant. Additionally, tree-boosting methods, LightGBM and XGBoost, showed no substantial difference in prediction accuracy compared to GCN, GATv2, and MPNN. Aside from these comparisons, most of the other methods demonstrated relatively significant differences in their solubility prediction capabilities.

For the imbalanced classification problem of EUOS/SLAS, the models we explored achieved a best public QCK score and private QCK score of 0.141 and 0.121, respectively. While the GCN model yielded the highest public QCK score, its performance in terms of the private QCK score was surpassed by both 1DCNN and XGBoost. However, the methods were not implemented in a competitive environment. Although the 1DCNN achieved a higher private score, its public score was not the highest, so it would not have been chosen as the final model submission in an actual competition setting. For this exercise, the results from the GNNs were not particularly impressive, so one might infer they would struggle to effectively generalize knowledge from such “quick-and-dirty” measurement data. It appears that most GNN methods are more sensitive to the inherent noise and outliers in the training data, limiting their stability and generalization capability.

In terms of the time costs incurred during the training, deployment, and prediction phases, more established tree-boosting algorithms such as XGBoost and LightGBM have a lower overhead. Selecting a subset of the most important features does not always guarantee improved performance. However, it can significantly reduce the time and computational resources required in scenarios such as real-time applications, working within resource-constrained environments, or when attempting to gain a preliminary understanding of a model’s performance potential.

When each type of model in data analysis brings its own set of strengths and capabilities, integrating multiple ML algorithms can leverage existing data from different perspectives, potentially improving predictive performance, as demonstrated by the winner of the first EUOS/SLAS solubility prediction challenge.^92^ However, this approach results in reduced interpretability, as a “black box” ensemble inevitably leads to heightened complexity, making the decision process difficult to trace and the extraction of meaningful insights problematic.

### Limitations and Future Work

We have evaluated some of the most popular ML methods of recent years using a considerably larger aqueous solubility data set than is typical of the field. Compared to the previously widely used cheminformatics approaches, our results suggest that that some of the most popular modern ML methods demonstrate superior performance in aqueous solubility prediction. However, the application of ML in solubility prediction still requires further development. Particularly, limitations in model interpretability, generalizability, adaptability to complex chemical systems, and in generating deep insights into molecular structures restrict the credibility and reliability of existing machine learning methods in scientific research and practical applications. Moreover, to make use of the information from both of the two main approaches to featurization, developing a hybrid DL model that employs both molecular graphs and molecular descriptors as inputs for predicting aqueous solubility is interesting to explore.

For ML methods that rely on descriptors as inputs, the performance of these models is inherently constrained by the quality of the selected descriptors. With the growth of high-throughput screening, there’s an increasing need for more refined molecular featurization techniques. However, several traditional molecular descriptors are found to lead to insufficiently accurate predictions for novel compounds or complex molecular systems. The incorporation of edge features in molecular graph representations has not significantly improved the performance in solubility predictions and may introduce additional noise. Therefore, for maximizing the utilization of information contained within the original data sets, modern molecular featurization methods, whether involving molecular graphs or descriptors, need to capture a wider array of chemical information such as quantum chemical properties, the three-dimensional structure of molecules, and stereochemical features. This study did not involve integrating 3D descriptors into the model. Although studies 18 years ago suggested that using 3D descriptors may not significantly improve solubility prediction,^93^ revisiting their potential in the future would still be valuable.

The regression data in this study cannot be directly used to predict classification data, and vice versa, the regression solubility data measured by CheqSol cannot be directly used to predict the classification data measured by nephelometry. However, it is worth exploring whether a model pretrained on the regression solubility data from CheqSol can improve the performance of the classification task through transfer learning by leveraging the learned relationships between molecular structure and solubility.

The predictive capability of ML models is, to some extent, positively correlated with the extent of coverage provided by larger training data sets, but the availability of high-quality data sets containing solubility data for complex druglike molecules from experimental sources is limited.^24,27,94^ Recently, generative artificial intelligence (AI) has rapidly emerged as a force transforming the way scientists and engineers approach cheminformatics challenges. It not only excels in generating text, images, and videos but also has potential applications in the design of small molecular compounds that are actively being explored.^95^ There is also the possibility of creating substantial quantities of synthetic data,^96^ increasing the diversity and size of training data sets, which could potentially improve the generalization capabilities of existing predictive solubility models. Further, variational autoencoders (VAEs) can learn latent representations of data, which can then be used as input for predictive models.

## Conclusions

The main objective of this work was to compare and evaluate the capabilities of various popular ML modeling methods and molecular representations in predicting aqueous solubility using a substantially larger data set of solubility data than commonly used in previous research. Compared to previously reported benchmarks, some of these ML methods showed incremental but significantly different improvements in predictive performance. Toward this objective, we have gained several insights.

GNN-based models that do not use 3D information, especially those with graph convolution and graph attention mechanisms, demonstrated promising performance when trained on high-quality solubility data sets. GAT and GCN, which do not directly process edge features, performed better than models considering edge features.

Models using molecular descriptors tend to be less sensitive to the inherent noise and errors in experimental solubility data and inherently offer more interpretability compared to models taking molecular graphs as inputs: tree boosting methods outperformed GNNs in tasks of predicting solubility data with higher average uncertainty in their solubility measurements. As suggested by Panapitiya et al.,^27^ this might be due to the ability of models using molecular descriptors to create better representations of molecules by mixing a wealth of information-rich structural descriptors without having to learn from the raw structure. These findings may, to some extent, be applicable to areas beyond solubility prediction, such as machine learning applications to ADMET properties.

In our importance analysis of the discussed set of 4480 commonly used molecular descriptors across different models, we concluded that about half were effective for solubility prediction, with approximately 800 contributing significantly to the prediction of aqueous solubility. The use of too many molecular descriptors with insufficient training data can lead to data sparsity, limiting model performance. By judicious feature selection, we reduced the computational resources needed in model training and deployment while ensuring expected predictive performance.

## Data Availability

The source code supporting the conclusions of this article are available in the GitHub repository, https://github.com/ECburx/MLSP.

## Supporting Information Available

Feature importance of each molecular descriptor across models, derived from 5-fold CV conducted for a range of ML methods (XLSX)
Results from ten runs of 5-fold CV and 50 individual predictions on various test sets for different ML methods (XLSX)
Predictions from single instance runs of various ML methods plotted against literature-sourced solubility values, and materials supporting the significance of differences in predictive performance among the ML methods (PDF)

## Abbreviations

ADME: absorption, distribution, metabolism, and excretion
AI: artificial intelligence
AttentiveFP: a graph-based neural network framework
ATS: autocorrelation of topological structures
CDK: chemistry development kit
CNN: convolutional neural network
CV: cross-validation
DL: deep learning
ECFP: extended-connectivity fingerprint
GCN: graph convolutional network
GAT: graph attention network
GATv2: a type of graph attention network which claims to overcome a confined form of attention that is computed by the GAT
GNN: graph neural network
LightGBM: a gradient boosting framework developed by Microsoft
MAT: molecular attention transformer
ML: machine learning
MLR: multiple linear regression
MPNN: message passing neural network
MW: molecular weights
QWK: quadratic weighted Cohen’s kappa
QED: quantitative estimation of drug-likeness
QSPR: quantitative structure–properties relationships
*R*^2^: coefficient of determination
RMSE: root mean square error
RNCG: relative negative charge
RPCG: relative positive charge
SHAP: Shapley’s additional explanation
SMILES: simplified molecular input line entry system
VAE: variational autoencoder
XGBoost: extreme gradient boosting
