# Supplementary material for: Revisiting the Application of Machine Learning Approaches in Predicting Aqueous Solubility
Source: ACS Omega. 2024 Jul 31;9(32):35209–22. doi: 10.1021/acsomega.4c06163 (PMC11325511; doi:10.1021/acsomega.4c06163)
Supplement: Supplementary file 3 — ao4c06163_si_003.pdf [file ao4c06163_si_003.pdf]

# Revisiting the Application of Machine Learning Approaches in Predicting Aqueous Solubility

Tianyuan Zheng,[*,†,¶] John B. O. Mitchell,[*,‡] and Simon Dobson[†]

†*School of Computer Science, University of St Andrews, St Andrews, Fife, UK*

‡*EaStCHEM School of Chemistry, University of St Andrews, St Andrews, Fife, UK*

¶*Current affiliation: Centre for Mathematical Sciences, University of Cambridge, Cambridge, Cambridgeshire, UK*

E-mail: tianyuanzhengac@gmail.com; jbom@st-andrews.ac.uk

Predictions from single instance runs of various ML methods plotted against literature-sourced solubility values, and materials supporting the significance of differences in predictive performance among the ML methods.

Table S3.1: Summary of results, represented as mean±SD of fifty runs (ten runs for CV), in terms of RMSE, $R^2$, and QCK, with the best results highlighted in bold.

| Test Set | Metric | 1DCNN | AttentiveFP | GAT | GATv2 | GCN | LightGBM | MPNN | XGBoost | $F$ | $p^a$ |
|---|---|---|---|---|---|---|---|---|---|---|---|
| 19SC1 | RMSE | 0.01 | 0.09 | 0.05 | 0.07 | 0.09 | 0.01 | 0.06 | 0.06 | 16.172 | 0.000 (***) |
| 19SC1 | $R^2$ | 0.01 | 0.13 | 0.07 | 0.11 | 0.13 | 0.01 | 0.10 | 0.10 | 17.270 | 0.000 (***) |
| 19SC2 | RMSE | 0.03 | 0.09 | 0.06 | 0.03 | 0.04 | 0.01 | 0.09 | 0.02 | 25.457 | 0.000 (***) |
| 19SC2 | $R^2$ | 0.02 | 0.08 | 0.05 | 0.13 | 0.03 | 0.01 | 0.08 | 0.01 | 7.854 | 0.000 (***) |
| 08SC | RMSE | 0.03 | 0.10 | 0.10 | 0.06 | 0.04 | 0.01 | 0.10 | 0.02 | 24.749 | 0.000 (***) |
| 08SC | $R^2$ | 0.03 | 0.09 | 0.09 | 0.05 | 0.03 | 0.01 | 0.12 | 0.02 | 26.532 | 0.000 (***) |

Note: *, **, and *** indicate increasing levels of statistical significance, with * being $p < 0.05$, ** $p < 0.01$, and *** $p < 0.001$; [a] The probability value $p$ measures the strength of evidence against the null hypothesis $H_0$ (there are no significant differences in the variances) in the hypothesis test.

Table S3.2: Based on the prediction results detailed in Supplemental Information 2 (SI2), we conducted the Brown-Forsythe ANOVA test to examine whether there were significant differences in the predictive performance of various ML methods across different test sets. The results showed that there were significant differences (***) in the predictions among at least one of the ML methods across all datasets. Consequently, further post hoc tests are required to determine the specific groups between which these differences are significant.

| Test Set | Metric | Brown $F$ | $p^a$ |
|---|---|---|---|
| 19SC1 | RMSE | 106.536 | 0.000 (***) |
| 19SC1 | $R^2$ | 100.204 | 0.000 (***) |
| 19SC2 | RMSE | 256.630 | 0.000 (***) |
| 19SC2 | $R^2$ | 119.138 | 0.000 (***) |
| 08SC | RMSE | 98.750 | 0.000 (***) |
| 08SC | $R^2$ | 105.096 | 0.000 (***) |

Note: *, **, and *** indicate increasing levels of statistical significance, with * being $p < 0.05$, ** $p < 0.01$, and *** $p < 0.001$; [a] The probability value $p$ measures the strength of evidence against the null hypothesis $H_0$ (there are no significant differences in the prediction capability across these ML methods) in the hypothesis test.

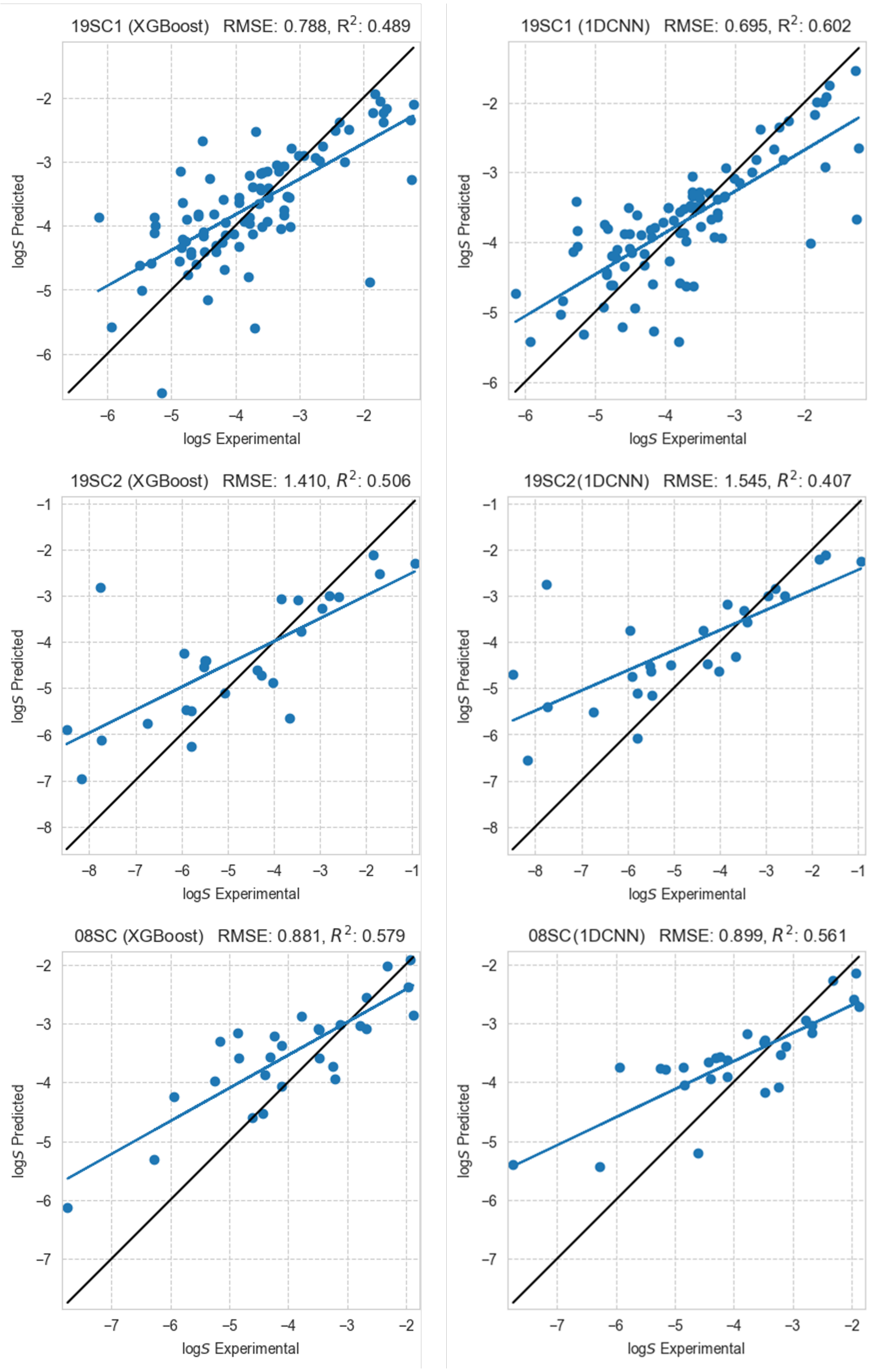


Figure S3.1: Predictions from XGBoost and 1DCNN are plotted against the literature log S values for compounds from the test sets 19SC1, 19SC2, and 08SC. The black diagonal line represents the perfect agreement between predicted and experimental solubility values, while the blue line indicates the best linear regression fit to these predictions.

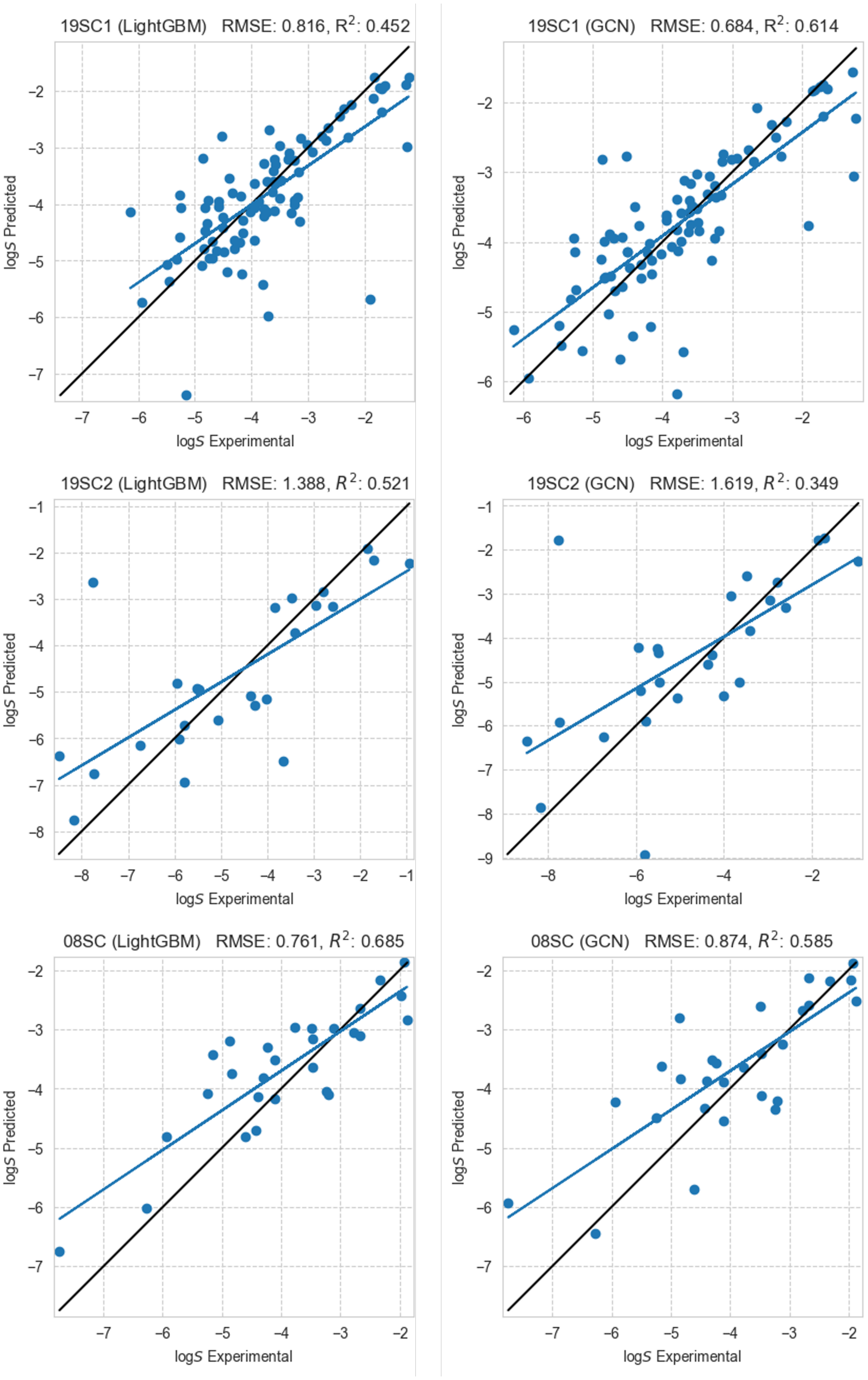


Figure S3.2: Predictions from LightGBM and GCN are plotted against the literature log S values for compounds from the test sets 19SC1, 19SC2, and 08SC. The black diagonal line represents the perfect agreement between predicted and experimental solubility values, while the blue line indicates the best linear regression fit to these predictions.

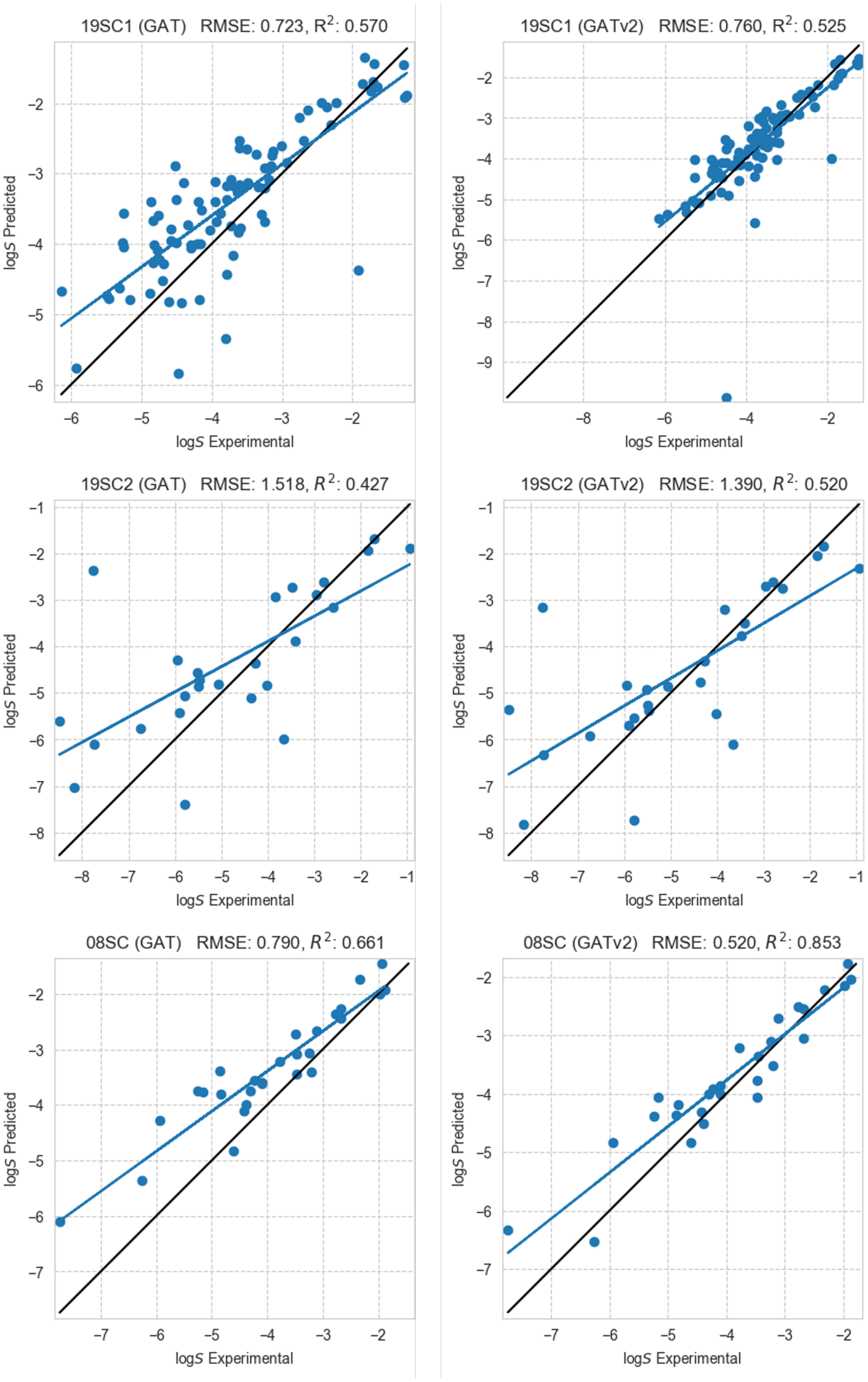


Figure S3.3: Predictions from GAT and GATv2 are plotted against the literature log S values for compounds from the test sets 19SC1, 19SC2, and 08SC. The black diagonal line represents the perfect agreement between predicted and experimental solubility values, while the blue line indicates the best linear regression fit to these predictions.

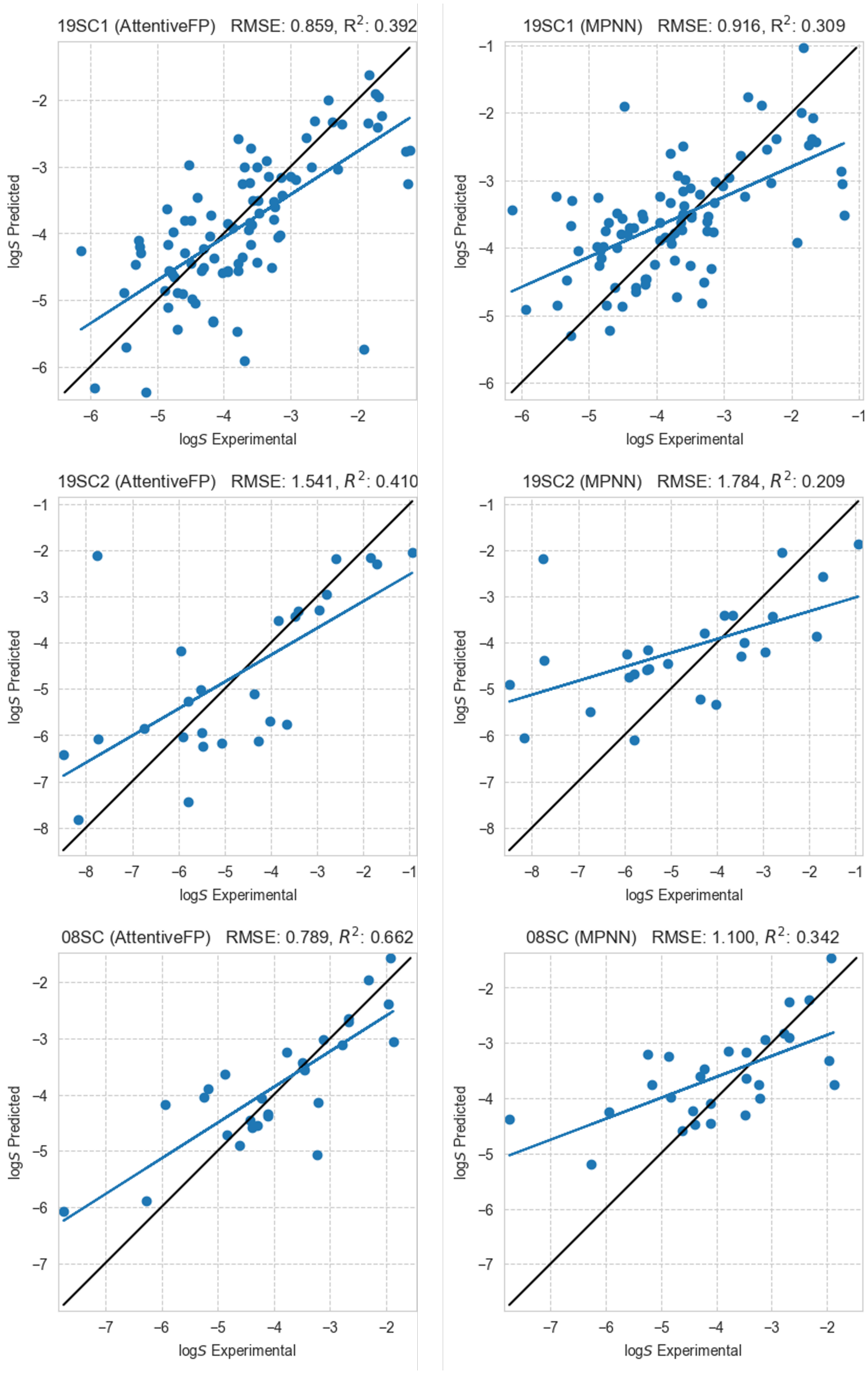


Figure S3.4: Predictions from AttentiveFP and MPNN are plotted against the literature log S values for compounds from the test sets 19SC1, 19SC2, and 08SC. The black diagonal line represents the perfect agreement between predicted and experimental solubility values, while the blue line indicates the best linear regression fit to these predictions.

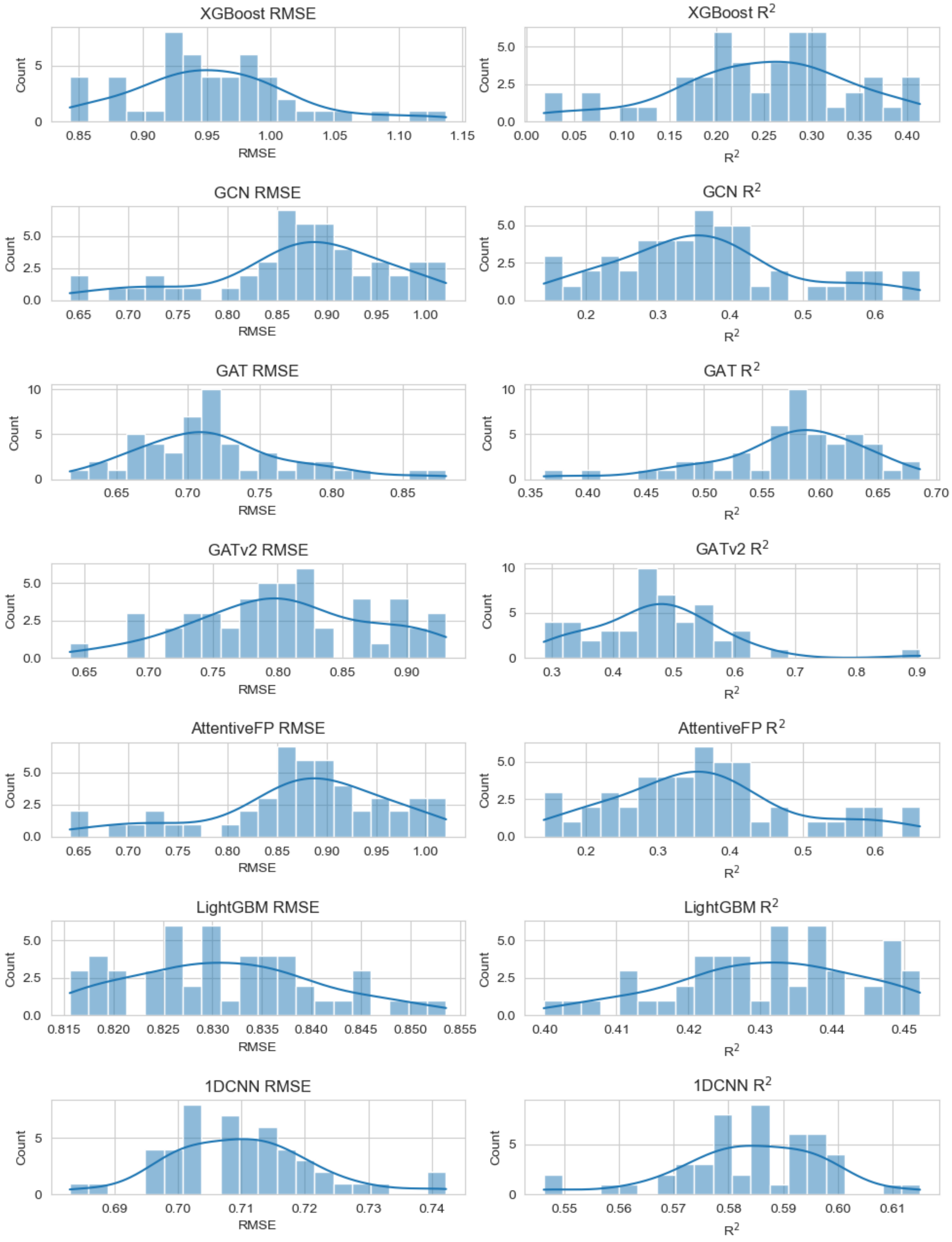


Figure S3.5: The distribution of RMSE and $R^2$ values for the prediction results on the 19SC1 test set, obtained from fifty training iterations using different ML modeling methods. The curves represent the kernel density estimates for these distributions.

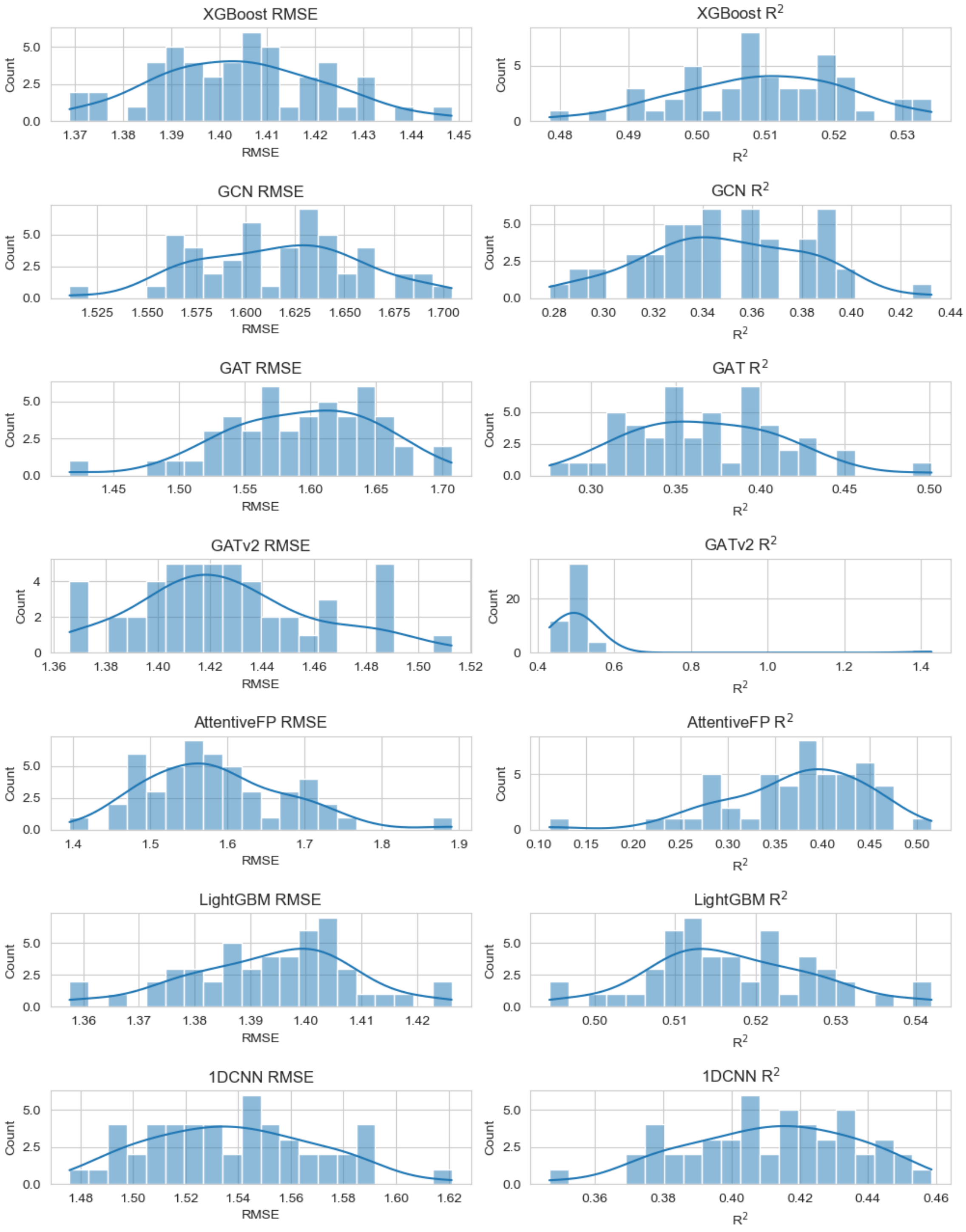


Figure S3.6: The distribution of RMSE and $R^2$ values for the prediction results on the 19SC2 test set, obtained from fifty training iterations using different ML modeling methods. The curves represent the kernel density estimates for these distributions.

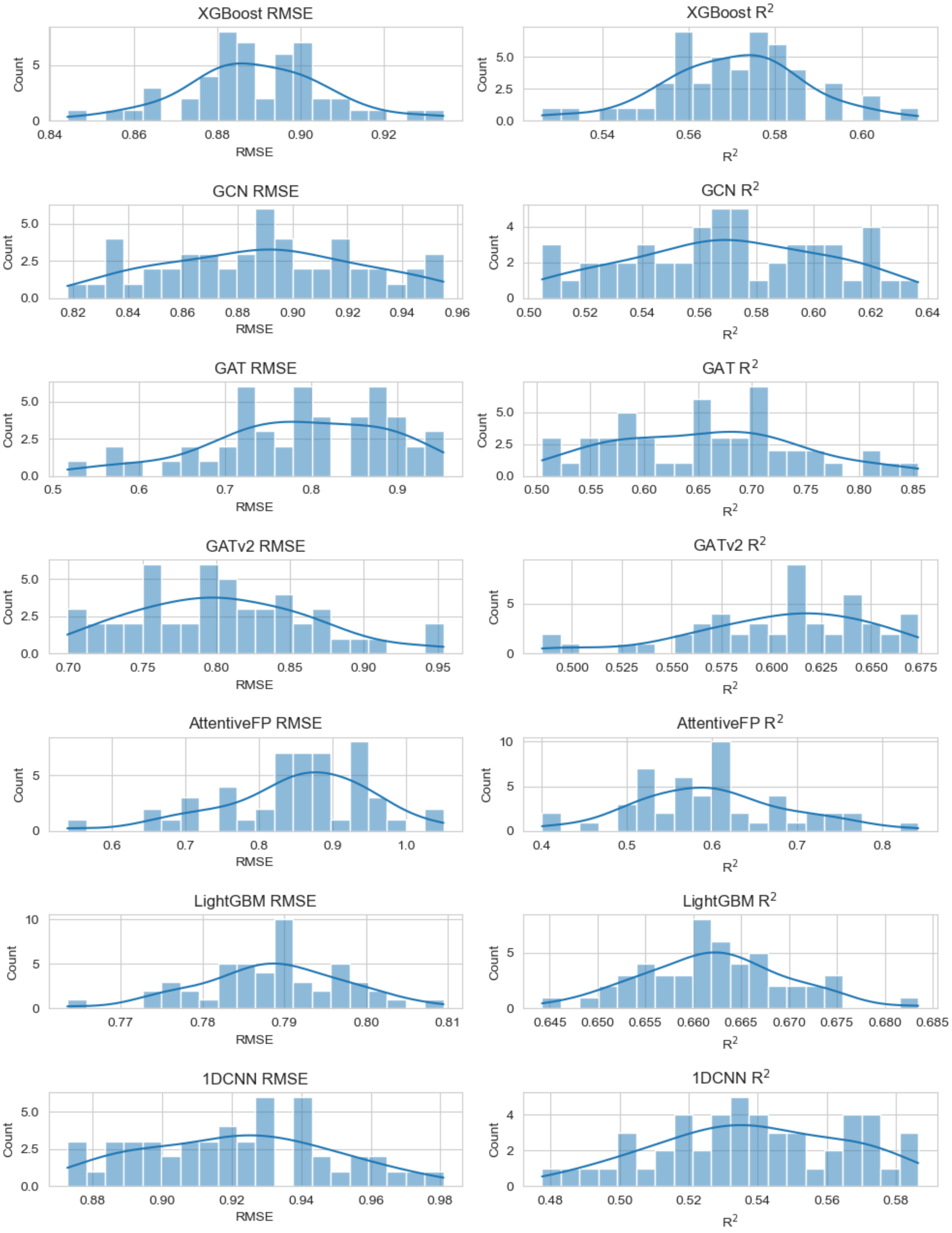


Figure S3.7: The distribution of RMSE and $R^2$ values for the prediction results on the 08SC test set, obtained from fifty training iterations using different ML modeling methods. The curves represent the kernel density estimates for these distributions.
